# P75NTR blockading inhibits Trem2+ M1 phenotype microglia activation and myelin damage following mild traumatic brain injury

**DOI:** 10.3389/fnins.2025.1641112

**Published:** 2026-01-07

**Authors:** Xu Li, Zhen Xu, Jing Fang, Hua-Dong Huang

**Affiliations:** 1 Department of Neurosurgery, The Second Affiliated Hospital, Zhejiang University School of Medicine, Hangzhou, China; 2 Department of Neurosurgery, The First Affiliated Hospital of Zhejiang Chinese Medical University (Zhejiang Provincial Hospital of Chinese Medicine), Hangzhou, China; 3 Department of Gerontology, The First Affiliated Hospital of Zhejiang Chinese Medical University (Zhejiang Provincial Hospital of Chinese Medicine), Hangzhou, China; 4 Department of Neurosurgery, The Affiliated Hospital of Youjiang Medical University for Nationalities, Baise, China

**Keywords:** mTBI, p75NTR, Trem2, M1 microglia, cognition deficit

## Abstract

The pathological basis underlying mild traumatic brain injury (mTBI)-induced long-term cognitive impairment is not fully understood. It is supposed that mTBI induces residential microglia activation rather than peripheral leukocyte infiltration to promote neuroinflammation, thus triggering myelin damage as well as cognitive impairment. The transformation of microglia towards a pro-inflammatory (M1 type) or anti-inflammatory (M2 type) state is critical for restraining the cerebral inflammatory response to acute or chronic insults. In addition to classical M1- and M2-like phenotypes, a specific subgroup of microglia, which is referred to as disease-associated microglia (DAM), the transition of which is regulated by triggering receptor expressed on myeloid cells 2 (Trem2), is also demonstrated to play a critical role in neurodegenerative diseases sharing similar pathological procedures to mTBI. The expression and function of p75 neurotrophin receptor (p75NTR) in microglia vary depending on the type and severity of the specific pathological stimuli. In the current study, we investigated whether peripheral leukocytes infiltrated the brain following mild traumatic brain injury (mTBI) using a CX3CR1- and CCR2-double transgenic reporter mouse model. We also examined whether M1- or M2-like microglia exhibited a disease-associated microglia (DAM) phenotype after mTBI, as indicated by their Trem2 expression. Then we explored the expression of p75NTR in M1- and M2-like phenotype microglia after mTBI and its modulating effects on the activation of Trem2 positive M1- and M2-like phenotype microglia, neuroinflammatory reaction, myelin damage, and cognitive performance. We found that most of the activated residential microglia after mTBI were Trem2 positive and p75NTR expression was significantly elevated in Trem2-positive M1-type microglia post-mTBI, correlating with increased pro-inflammatory cytokine release, demyelination, and cognitive deficits. Pharmacological blockade of p75NTR using the antagonist TAT-Pep5 suppressed M1 microglial activation, reduced neuroinflammation, and restored myelin integrity, leading to marked improvements in cognitive function. Mechanistically, p75NTR exhibited a cell-type-specific regulatory role in neuroinflammatory responses, potentially through interacting with Trem2 to modulate DAM-like microglia activation. These findings highlight p75NTR as a key mediator of mTBI-induced neuropathology and propose its inhibition as a novel therapeutic strategy to mitigate secondary neuroinflammation and cognitive decline.

## Introduction

Annually, about 61 million people worldwide suffer from traumatic brain injury (TBI). Most of these injuries are mild, namely mild traumatic brain injury (mTBI), accounting for approximately 80% of all TBI cases. Chronic cognitive impairment is one of the most common sequelae after mTBI, which can last months to years after the initial trauma and seriously affects patients’ normal studies, work, and daily life (Nance et al., 2009). Unlike moderate and severe TBI, the pathological basis underlying mTBI-induced long-term cognitive impairment is confusing and poorly characterized. In moderate to severe TBI, primary and secondary injuries lead to neuronal death and white matter injury (WMI), characterized by demyelination and/or axonal damage, which play foundational roles in the pathophysiological processes. In the context of mTBI, demyelination resulting from oligodendrocyte loss rather than axonal damage is thought to be the main pathological basis underlying WMI, which has been closely linked to cognitive impairment (Mahoney et al., 2022; Mierzwa et al., 2015).

Neuroinflammation is a central component of the secondary injury response to TBI and has a strong influence over long-term neurological outcomes following TBI. A neuroinflammation reaction following TBI is primarily mediated by activated resident microglial cells and recruited peripheral immune cells such as neutrophils and monocytes (Cederberg and Siesjö, 2010). The early infiltration of peripheral leukocytes after TBI depends on the severity of acute blood–brain barrier (BBB) damage determined by the degree of initial mechanical violence and the following neuroinflammation reaction. Infiltrating leukocyte counts increase in proportion to the severity of TBI. In mTBI, resident microglia might play a more critical role in acute neuroinflammation reaction and secondary injury due to the less infiltrated leukocytes (Trahanas et al., 2015). Microglia show both a detrimental pro-inflammatory (M1 type) effect and a beneficial anti-inflammatory (M2 type) effect after TBI (Younger et al., 2019). The M1-like phenotype with specific hallmarks, such as CD16 and CD32, usually secretes pro-inflammatory factors, such as tumor necrosis factor-α (TNF-α), interleukin-1*β* (IL-1β), and inducible nitric oxide synthase (iNOS). On the contrary, the M2-like phenotype, labeled by the molecular marks of CD206 and arginase 1 (Arg-1), is thought to release anti-inflammatory mediators including interleukin-10 (IL-10) and transforming growth factor-β (TGF-β) (Chan et al., 2000). Generally, in neurodegenerative and other CNS pathological processes characterized by uncontrolled neuroinflammation reaction, the number of pro-inflammatory M1-like phenotypes is far more than the M2 type. Reducing M1 microglia-induced inflammatory responses is thought to be a promising treatment for mitigating neuronal damage (Hansen et al., 2018; Dong et al., 2019; Liu et al., 2019).

Beyond classical M1- and M2-like phenotypes, massive transcriptomic analysis has uncovered that microglia can transition to various states, displaying distinct intrinsic characteristics and performing unique functions under specific conditions (Stratoulias et al., 2019; Mendes and Majewska, 2021). Among them, a specific subgroup of microglia, which is referred to as disease-associated microglia (DAM), the transition of which is regulated by triggering receptor expressed on myeloid cells 2 (Trem2), is demonstrated to play critical roles at various stages of neurodegenerative diseases in the central nervous system (CNS) (Samant et al., 2024; Rangaraju et al., 2018). Trem2, which serves as a receptor for a multitude of ligands, enhancing their phagocytic activity, has emerged as a critical modulator of microglial activity. When DAM is involved in neuroinflammation, the Trem2 signal plays bidirectional roles. On more rare occasions, Trem2 signal activation might modulate defense mechanisms, but more often its dysregulation will significantly promote neuroinflammation, which presents great challenges for therapeutic targets (Stratoulias et al., 2019; Samant et al., 2024). The p75 neurotrophin receptor (p75NTR), which belongs to the TNF receptor superfamily, can modulate a variety of biological functions through its binding to pro-neurotrophins with high affinity. Although P75NTR can directly bind to mature neurotrophins, due to the very low affinity their binding is too loose to induce a downstream reaction. Instead, the monomer condition of p75NTR performs its multiple biological roles in neuronal survival, neurite outgrowth, and axonal regeneration through interactions with its multiple coreceptors (tropomyosin-related kinase receptors, Trks) to form p75NTR-Trks dimers and induce Trk signaling (Chan et al., 2000). Under physiological conditions, the expression of P75NTR in CNS gradually downregulates as the brain matures; in a fully developed brain the expression of p75NTR is restricted to a few regions (Friedman, 2000; Schor, 2005; Yan and Johnson, 1988). However, under various CNS pathological stimuli (Ibáñez and Simi, 2012), a striking upregulation of p75NTR, along with its ligand pro-NGF, will be quickly elicited in the adult brain (Beattie et al., 2002; Nykjaer et al., 2004). The microglial expression and its biochemical function in microglia-induced inflammation have not been fully explored. The expression of P75NTR in microglia is influenced by the living matrix and environment. Microglial cells *in vitro* were also found to express p75NTR (Shen et al., 2013; Srinivasan et al., 2004), but their expression *in vivo* is debatable and might be determined by the specific type of CNS lesions. In a rodent model of severe TBI, microglial cells did not express P75NTR (Lee et al., 2016). In a mouse ischemic model induced by middle cerebral artery occlusion, both the resident microglia and peripheral-derived macrophage expressed p75NTR as early as the first 24 h after ischemic induction (Lambertsen et al., 2007). In a mouse model of multiple sclerosis (MS), where the pro-inflammatory microglia and macrophage causes oligodendrocyte loss and demyelination, p75NTR expression was also upregulated in these immune cells (Dowling et al., 1999). In one published study from our team, we demonstrated the striking upregulation of p75NTR expression in residential microglia in a SAH model, where the p75NTR antagonist TAT-Pep5 efficiently reduced the activated microglia number, neuroinflammation, and early brain injury (Xu et al., 2019). The pathological cascades of mTBI are not totally equal to severe TBI, stroke, or neurodegeneration; neuroinflammation is one of their common pathogeneses and it is intriguing to explore the microglial expression and function of p75NTR under the pathological condition of mTBI.

Therefore, we designed this study to explore the nature of P75NTR expression in mTBI-activated microglia and the effects of P75NTR blockading on Trem2-positive microglia activation, neuroinflammation, and cognition performance after mTBI.

## Materials and methods

### Animals

Adult female Cx3cr1-GFP/^+^Ccr2-RFP/^+^ double transgenic mice on a C57BL/6 background (average age: 65 ± 5 days; average weight: 23 ± 2.1 g) and wild-type C57BL/6 mice (average age: 65 ± 5 days; average weight: 24 ± 2.3 g) were used in this study to control for sex as a biological variable. Animal use and care was approved by the Animal Care and Use Committee of Zhejiang Chinese Medicine University, in accordance with all relevant national laws. Animals were housed in filter-top cages (5/cage) and fed with a regular criterion diet, with a 12-h light/dark cycle. Constant temperature (23 ± 1 °C) and humidity (65 ± 5%) were maintained, with food and water provided *ad libitum*.

Cx3cr1GFP/^+^Ccr2RFP/^+^ double transgenic mice were established as in our previous study and others (Xu et al., 2019; Morganti et al., 2015; Saederup et al., 2010; Umekawa et al., 2015). Cx3cr1GFP/GFP and Ccr2RFP/RFP mice were purchased from the Jackson Laboratory (Bar Harbor, ME). After genotyping by PCR, first-generation littermates were used for this experiment.

### Midline fluid percussion injury and drug administration

Equal numbers of Cx3cr1GFP/^+^Ccr2RFP/^+^ double transgenic mice were randomly assigned into sham and mTBI groups (*n* = 4). Wild C57BL/6 mice were randomly assigned to mTBI (*n* = 8), sham injury (*n* = 8), or mTBI^+^ TATPep5 (*n* = 8). The individual performing the mTBI surgery was blinded to other experimental procedures. For each animal, the group assignment was unknown until data collection was finished.

The midline fluid percussion injury (FPI) method was adopted for producing the mice mTBI model, as described previously (Lifshitz et al., 2016; Rowe et al., 2016). Animals were anesthetized with isoflurane and the scalp was incised beside the midline. A circular craniotomy about 3 mm was conducted with a mini drill at the center of the sagittal suture midway between the bregma and lambda. During the surgery, the dura and brain tissue were carefully protected. An injury cap was remolded to fit within the craniotomy, then an appropriate dose of cyanoacrylate gel and methyl-methacrylate (Hygenic Corp., Akron, OH, United States) was used to tightly affix the cap over the craniotomy. After surgery, the incision was sutured, and a suitable dosage of lidocaine ointment and topical bacitracin were administered. Body temperature was monitored by rectal thermometers and sustained with isothermal heating pads. Before the induction of mTBI, the injury-hub assembly was filled with normal saline to check the watertightness and integrity of dura. Then a fluid percussion device (Custom Design and Fabrication, Virginia Commonwealth University, Richmond, VA, United States) was attached to release the pendulum onto the fluid-filled cylinder (mean atm: 1.98 ± 0.04). The severity of the injury was assessed using the forearm fencing reaction and the recovery time of the righting reflex, which was about 321 ± 24 s in mTBI mice and less than 10 s in sham-injured animals (Hosseini and Lifshitz, 2009). No animals suffered apnea or death and the post-operation health condition of each animal was estimated for 2 days. The body weight of each animal was maintained above 95% of their pre-operative weight. After induction of mTBI, animals were intraperitoneally administrated with the p75NTR specific antagonist TAT-Pep5 [H-YGRKKRRQRRR-CFFRGGFFNHNPRYC-OH] (Chemocentryx) dissolved in PBS (1.7 mg/kg) or vehicle (PBS) daily (Alder et al., 2016; Ansar et al., 2011).

### Histological analysis

On 2 and 4 weeks after mTBI, mice were anesthetized with a lethal dose of isoflurane. Then mice were transcardially perfused with ice cold 0.1 mmol PBS and 4% paraformaldehyde (PFA). Brains were removed from the skull and transferred into 4 °C formaldehyde (PBF) solution (4%). After a 24 h fixation time, brains were soaked in different concentrations of sucrose solution for about 72 h, then frozen with powdered dry ice. Brians were cut to 10 μm thick sections from bregma with 200 μm intervals and collected on glass slides for staining.

For immunohistochemistry, after being pretreated with 0.1% Triton X-100, sections were incubated with the following primary antibodies in a 4 °C incubator for 24 h (1:500 anti-DsRed polyclonal antibody, Clontech; 1:200 anti-CD16/32 antibody, Abcam; 1:400 anti-CD206 antibody, Abca; 1:500 anti-Iba-1 antibody, Wako; 1:200 anti-MBP antibody, Abcam;1:200 anti-Trem2 antibody, Abcam and 1:300 anti-p75NT antibody, Abcam). Then the corresponding secondary antibody was added and incubation continued for another 1 h at room temperature. Immunohistochemical images were captured in a blinded way under a confocal laser-scanning microscope (Leica SP8). Image Pro Plus 6.0 Software (MediaCybernetics, Bethesda, MD) was used for image analysis and quantification of immunofluorescent. For each section, observers blinded to the experiment randomly chose 10 vision fields without overlap for calculating, and the total number of targeted cells were counted by the mean number of immunopositive cells in the 10 chosen scope. For each animal, eight sections were chosen from bregma to lambda with a 200 μm interval. The total number of targeted cells were counted by the final average numbers of immunopositive cells in eight sections.

### Real-time quantitative RT-PCR

Total RNA of each inflammatory factor were determined by RT-qPCR (Wang et al., 2013). TRIzol reagent (Invitrogen) was used to extract RNA from brain tissues. After confirming the quality of extracted RNA by agarose gel electrophoresis and the spectrophotometric method, RNA pretreated with DNase was reverse-transcribed to cDNA using SuperScript II Reverse Transcriptase (Invitrogen). A CFX96 Real-time PCR detection system (Bio-Rad) was used to perform RT-qPCR with 20 ng cDNA and the following primers. Specific primer sequences (Genscript Biological Technology Company, Nanjing, China) are listed as follows: TNF-α primer [50′-AAATGGGCTCCCTCTCATCAGTTC-30′ (forward) and 50′-TCTGCTTGGTGGTTTGCTACGAC-30′ (reverse)], IL-1β primers [50′ -CACCTCTCAAGCAGAGCACAG-30′ (forward) and 50′-GGGTTCCATGGTGAAGTCAAC-30′ (reverse)], and GAPDH primers [5′-ACAGCAACAGGGTGGTGGAC-3′ (forward) and 5′-

TTT GAGGGTGCAGCGAACTT-3′ (reverse)]. Line-Gene software was used to determine each mRNA level by the 2^−ΔΔCT^ method and checked against the GAPDH gene.

### Western blot

The ipsilateral cortex samples from the sham and mTBI brains were homogenized on ice using 400 μL of modified RIPA buffer (0.1% SDS, 50 mM Tris-HC, 1 mM EDTA, 1% Triton X-100, 100 mM NaCl and 1% sodium deoxycholate). The protease and phosphatase inhibitors (Roche) were added into lysate and this was then agitated for 2 h, centrifuged at 16,000 g for 15 min, and stored at −30 °C prior to assay. Following the protein level assay, the supernatant was suspended in Laemmli sample buffer. After denaturation at 95 °C for 5 min, proteins were separated on tris-glycine 4–15% acrylamide gels and transferred to PVDF membranes soaked in 5% non-fat milk in 0.05% PBS-Tween 20 for 2 h. The proteins of iNOS and β-actin were tested by anti-iNOS (1:200, Abcam) and anti-β-actin (1:500, Abcam), respectively. The secondary HRP-coupled antibody was added and incubated for 1 h at room temperature followed by the ECL plus reagent (Santa Cruz). After rinsing several times with TBS-Tween, the MiVnt image analysis system (Bio-Rad, Carlsbad, CA, United States) was used to evaluate the densities of the bands.

### ELISA analysis

Sample tissue was homogenized in T-Per reagent containing protease inhibitors (Thermo Scientific) and the supernatants were collected by centrifugation for protein determination with ELISA Kits (Invitrogen) according to the instructions.

### Cognitive function evaluation

The cognitive function assessments were performed at 2- and 4-weeks post mTBI by investigators blinded to treatment allocation. The novel object recognition (NOR) task, novel object location (NOL) task, and temporal order object recognition (TOR) task were used to test short-term recognition memory, long-term spatial memory, and temporal working memory, respectively, as described (Griffiths et al., 2022; Ennaceur et al., 1996; Barker et al., 2007). The NOR tasks took place in a squared box (31.5 × 31.5 cm) and environmental noises were covered with white noise (~46 Db). After acclimating to the arena for 3 min, animals were presented with two objects (O1 and O2) in opposite corners of the box (5 min) in the sample trial.

Then an object (O2) was replaced by a novel object (O3) and, 4 h later, mice were returned to the arena. Normal mice explored the novel object (O3) more than the familiar object (Ennaceur et al., 1996). The test trial of NOR served as the sample trial. The NOL tasks took place in the same box. After 24 h, object O1 was moved to an adjacent corner of the arena, while object O3 was kept in the same place. Normal mice explored the object O1 in the novel location more than the unmoved object O3. The TOR task tested temporal working memory by the ability to recognize the order of objects presented over time (Barker et al., 2007). The cognitive framework was established by two sample trials, in which mice explored two copies of an object and a separate pair of identical objects for 5 min, respectively, with a 3 min break between them. After 5 min, the test trial was followed with one of each item present. Normal mice explored the initial object rather than the more recent object. Exploration of an object being within ~2 cm of the object was defined as the nose. For all tasks, differences in the time of exploring were recorded. The exploration time of the target object/exploration time of both objects was defined as the discrimination ratio. Normal mice explored the target object more than the original object, resulting in a discrimination ratio above 0.5 (Barker et al., 2007). A discrimination ratio of 0.5 suggested equal exploration of the object and equivalence to chance performance. Ethovision software (Noldus, Leesburg, VA, United States) was used to track and record the trials.

### Statistics

The data were reported as the means ± standard error of mean. IBM SPSS software (version 29.0) was used to analyze the data. Graphs were produced by Graphpad Prism software. Data analysis was performed using one-way analysis of variance (ANOVA) followed by Tukey multiple comparison *post-hoc* analysis. The Spearman correlation was also determined. *p* < 0.05 was considered to indicate a statistically significant difference.

## Results

### Resident microglia activated after mTBI

**Figure 1:**
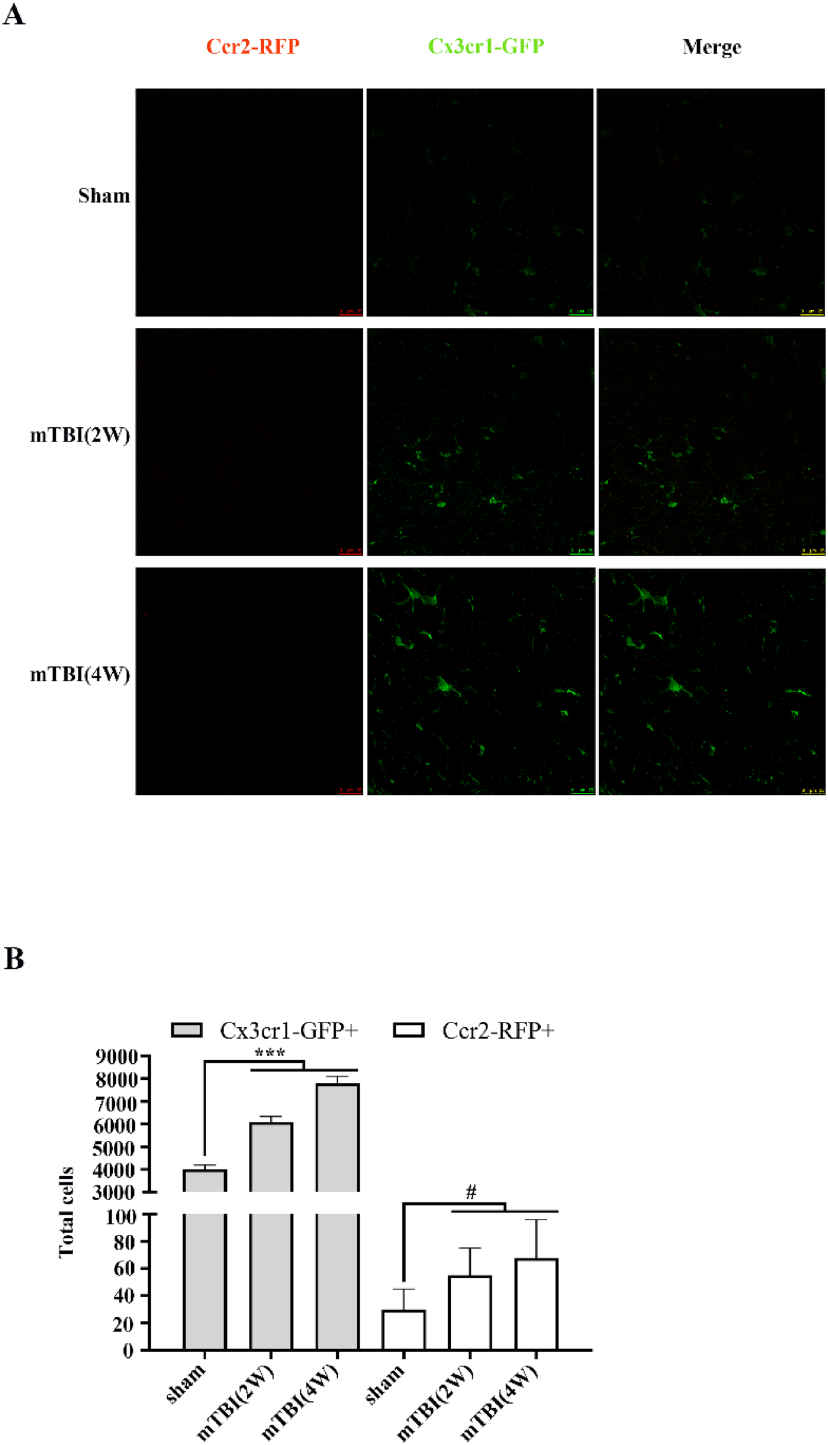
Resident microglia activated after mTBI. **(A)** Representative images of Cx3cr1-GFP+ (green) and Ccr2-RFP+ (red) staining cells in sham and injured brains at 2- and 4-weeks post mTBI. **(B)** Cx3cr1-GFP+ cells increased in white matter surrounding the injured cortex from 2 to 4 weeks after mTBI, while Ccr2-RFP+ cells were almost undetected. Scale bars: 25 μM. Data were expressed as mean ± SEM (*n* = 4/group, ^***^p < 0.001 versus sham, ^#^*p* > 0.05 versus sham).

As suggested by Trahanas et al. (2015), resident microglia rather than infiltrated leukocytes might be involved in the acute neuroinflammation reaction in mTBI. We first studied the numbers of activated resident microglia and blood-derived macrophage in brains with mTBI. The standard antibody markers (e.g., Iba1) used to stain microglia do not distinguish between resident microglia and peripheral macrophages, leading to uncertainty on their origins. A unique CX3C-chemokine receptor 1 (Cx3cr1) and chemokine receptor 2 (Ccr2) double transgenic reporter mouse was used to distinguish microglia from peripheral CNS macrophage by examining the expression of Cx3cr1-GFP and Ccr2-RFP on cell surfaces at 2 and 4 weeks post mTBI (Xu et al., 2019; Morganti et al., 2015; Umekawa et al., 2015). Cx3cr1-GFP^+^ cells existed in sham brains and their numbers in the ipsilateral white matter of injury increased sharply at 2 weeks post mTBI, then further increased at 4 weeks (Figures 1A,B). Ccr2-RFP^+^ cells were barely apparent in the sham brains. Although their numbers also increased with time after mTBI, their differences were statistically meaningless (Figures 1A,B). These results indicated that peripheral macrophages did not infiltrate into the brain and resident microglial cells activated and induced neuroinflammation reaction after mTBI.

### Microglial expression of p75NTR following mTBI

**Figure 2:**
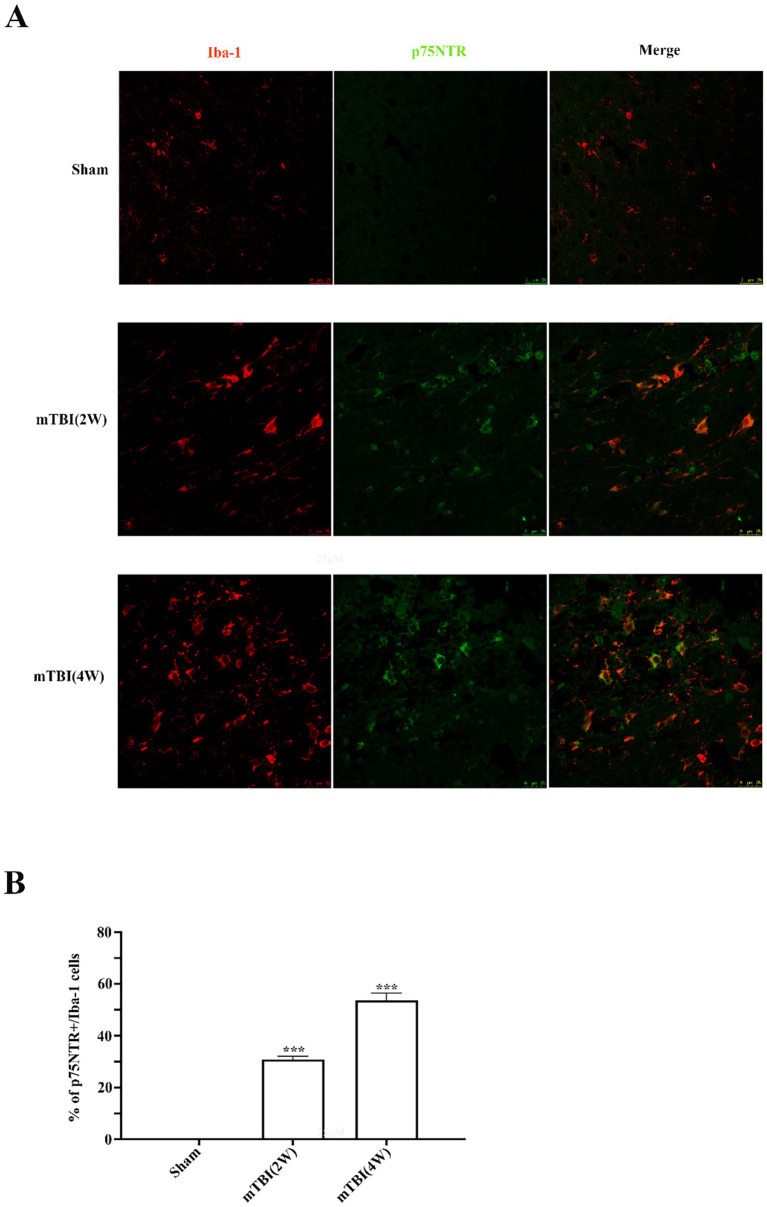
Microglial expression of p75NTR following mTBI. **(A)** Representative images of p75NTR+ (green) and Iba-1+ (red) staining cells in sham and injured brains at 2- and 4-weeks post mTBI. **(B)** mTBI induced p75NTR expression in Iba-1+ microglial cells from 2 to 4 weeks post mTBI. Scale bars: 25 μM. Data were expressed as mean ± SEM (*n* = 8/group, ^***^p < 0.001 versus sham).

As proven by previous experiments, the peripheral macrophage did not infiltrate into the brain post mTBI. Thus, in the following experiments, we used the wild-type mouse and regular antibody to mark microglia for further study. We tested whether p75NTR was expressed in microglia in response to mTBI using double immunofluorescent staining of p75NTR and microglial marker Iba-1. We found Iba-1 positive microglia increased at 2 weeks in wild brain mice after mTBI and further increased at 4 weeks post mTBI, indicating mTBI stimulated residential microglia activation in a time-dependent way. In wild sham brain mice, p75NTR was not detected in Iba-1 immunopositive cells. After mTBI, p75NTR was observed in a great number of Iba-1positive cells as early as 2 weeks and up to 4 weeks (approximately 30.8% at 2 weeks and 53.6% at 4 weeks) (Figures 2A,B). These results demonstrated that mTBI induced lasting upregulation of p75NTR in activated microglial cells.

### P75NTR modulated Trem2+ M1 phenotype microglia activation after mTBI

**Figure 3:**
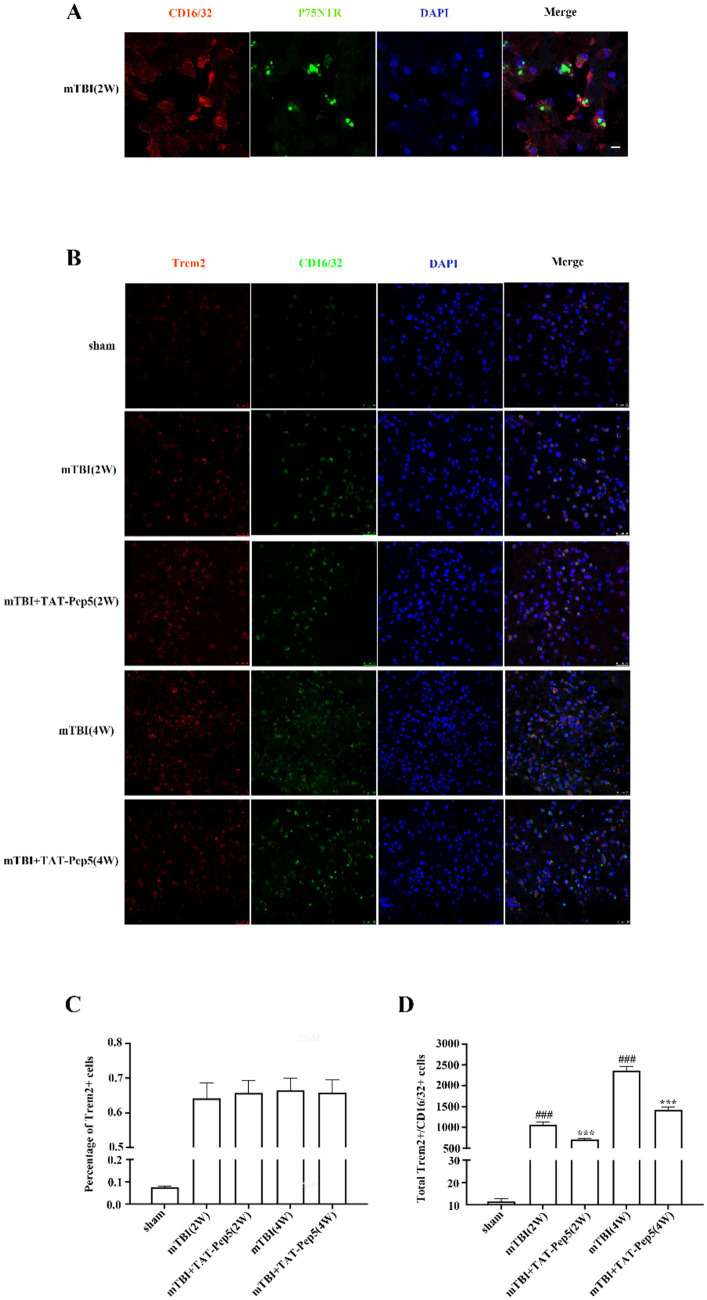
P75NTR modulated Trem2+ M1 phenotype microglia activation after mTBI. **(A)** Representative images of p75NTR (green) and CD16/32 (red) staining cells in injured brains at 2 weeks post mTBI. Scale bars: 8 μM. **(B)** Representative images of CD16/32 (green) and Trem2 (red) staining cells in injured brains treated with vehicle or TAT-Pep5 at 2- and 4-weeks post mTBI. Scale bars: 25 μM. **(C)** The percentage of Trem2+ cells in CD16/32^+^ microglia with or without TAT-Pep5 treatment. Data were expressed as mean±SEM (*n* = 8/group). **(D)** CD16/32^+^/Trem2+ cells increased in white matter surrounding the injured cortex from 2 to 4 weeks after mTBI and reduced by TAT-Pep5 treatment. Data were expressed as mean±SEM (*n* = 8/group, ^***^p < 0.001 versus vehicle, ^###^*p* < 0.001 versus sham).

The Iba-1+ microglia may include both M1- and M2-like phenotypes. To specifically evaluate the effect of p75NTR modulation on M1 or M2 type microglial activation after mTBI, we firstly explored the p75NTR expression on these two microglial cells by co-labeling CD206 or CD16/32 with p75NTR in the white matter surrounding the mTBI-affected regions. Results from double immunofluorescent staining demonstrated that p75NTR could hardly be observed in CD206-positive cells (Supplementary Figure 1A) but was highly expressed in the CD16/32-positive cell population in the perilesional area (Figure 3A), indicating p75NTR upregulated in M1 but not M2-like cells after mTBI.

To date, the activation and etiology of DAM are only understood within the context of neurodegenerative diseases such as Alzheimer’s Disease (AD), Parkinson’s disease (PD), and Multiple Sclerosis (MS). Many studies have suggested a link between mTBI and neurodegenerative diseases, specifically AD and chronic traumatic encephalopathy (CTE) (Fakhran and Alhilali, 2014; Schofield et al., 1997; Lee et al., 2013). We co-labeled CD206 or CD16/32 with the typical DAM marker Trem2 in the white matter surrounding the mTBI-affected regions to determine if mTBI-activated M1 and M2 type microglia had DAM features. Results showed that CD16/32 and CD206 positive cells could be observed in sham brains at a very low level, but none of them were labeled with Trem2. After mTBI, CD16/32 and CD206 positive cells strongly increased at 2 weeks and further increased at 4 weeks, and most of them were also synchronously Trem2 positive (approximately 64.1 and 66.5% of CD16/32^+^ cells at 2 and 4 weeks, 72.6 and 75.2% of CD206^+^ cell at 2 and 4 weeks) (Figure 3C; Supplementary Figure 1C). These results indicated that mTBI stimulated M1 and M2 microglia activation with a DAM feature. We then examined whether p75NTR blockade would affect the Trem2+ M1 and M2-like microglia activation by intraperitoneally injecting p75NTR-specific antagonist TAT-Pep5. Results showed that, compared with vehicle, TAT-Pep5 injection significantly reduced the total number of CD16/32 and CD16/32^+^/Trem2+ microglia at 2 and 4 weeks post-mTBI (Figures 3B,D). On the other hand, the number of CD206 and CD206^+^/Trem2+ microglia were not significantly changed by the p75NTR-specific antagonist TAT-Pep5 (Supplementary Figures 1B,D). The percentage of Trem2 positive CD16/32^+^ and CD206^+^ cells were not significantly changed by TAT-Pep5 treatment (approximately 65.7 and 65.8% of CD16/32^+^ cells at 2 and 4 weeks, 73.2 and 74.3% of CD206^+^ cell at 2 and 4 weeks) (Figure 3C; Supplementary Figure 1C).

In summary, these results indicate that microglia activated following mTBI exhibit DAM markers similar to those found in neurodegenerative conditions. Furthermore, p75NTR may play a critical role in promoting the proinflammatory activation of Trem2-positive M1-like microglia.

### p75NTR blockade reduced proinflammatory cytokine levels after mTBI

**Figure 4:**
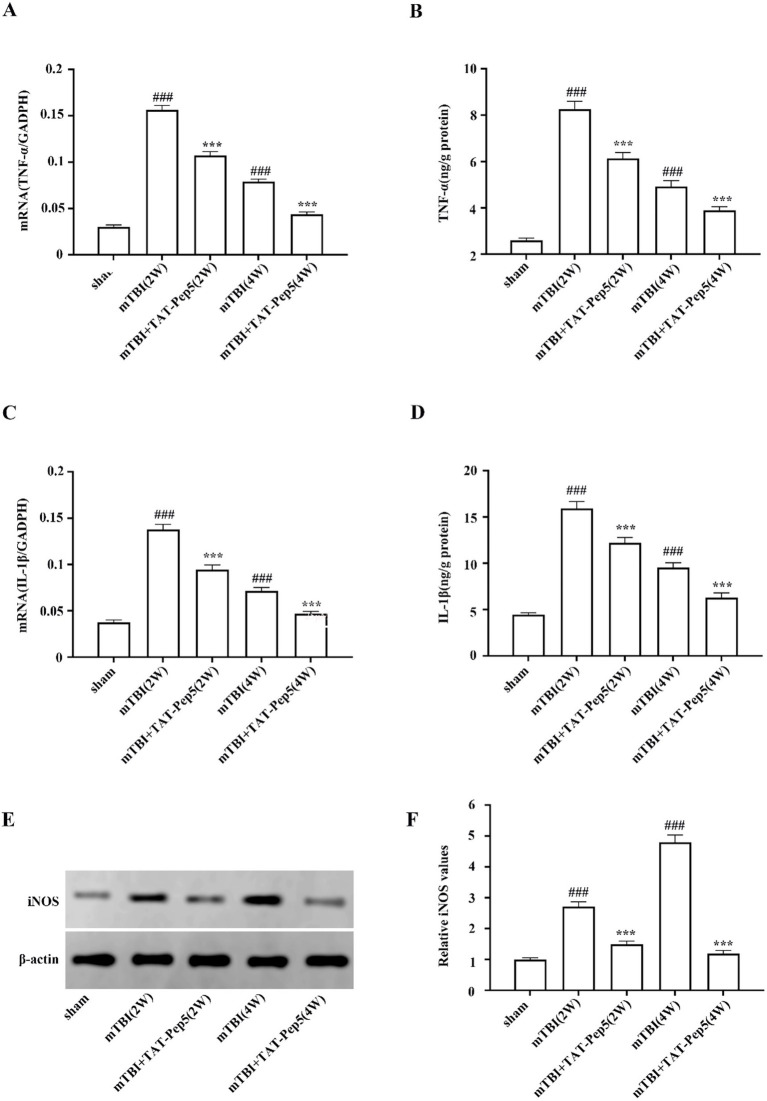
p75NTR blockade reduced proinflammatory cytokine levels after mTBI. **(A,C)** RT-qPCR analysis showed that mRNA expressions of TNF-α and IL-1β were upregulated after mTBI and downregulated by TAT-Pep5 treatments. **(B,D)** Enzyme-linked immunosorbent assay showed the protein expression of TNF-α and IL-1β were upregulated after mTBI and downregulated by TAT-Pep treatments. **(E,F)** Western blot analysis showed the protein expression of iNOS was upregulated after mTBI and downregulated by TAT-Pep treatments. Data were expressed as mean ± SEM (*n* = 8/group, ^***^p < 0.001 versus vehicle, ^###^*p* < 0.001 versus sham).

Compared to M2-like phenotype microglia, the M1-like microglia tend to release proinflammatory factors in the insulted brain. By determining the mRNA and protein levels of proinflammatory factors such as TNF-α, IL-1β, and iNOS after p75NTR blockading, we explored the functions of p75NTR on mTBI-induced neuroinflammation. RT-qPCR assay showed that mTBI induced significant upregulation of TNF-α, IL-1β, and iNOS mRNA. Accordingly, the protein levels of TNF-α, IL-1β, and iNOS were also increased and lasted at least 4 weeks after injury, as demonstrated by Elisa and WB assay (Figures 4A–E). On the contrary, p75NTR blocking by specific antagonist TAT-Pep5 showed significant anti-inflammatory effects, as demonstrated by the attenuated cerebral mRNA and protein levels of TNF-α, IL-1β, and iNOS at 2 and 4 weeks post-injury (Figures 4A–F).

### Inhibition of p75NTR alleviated myelin damage after mTBI

**Figure 5:**
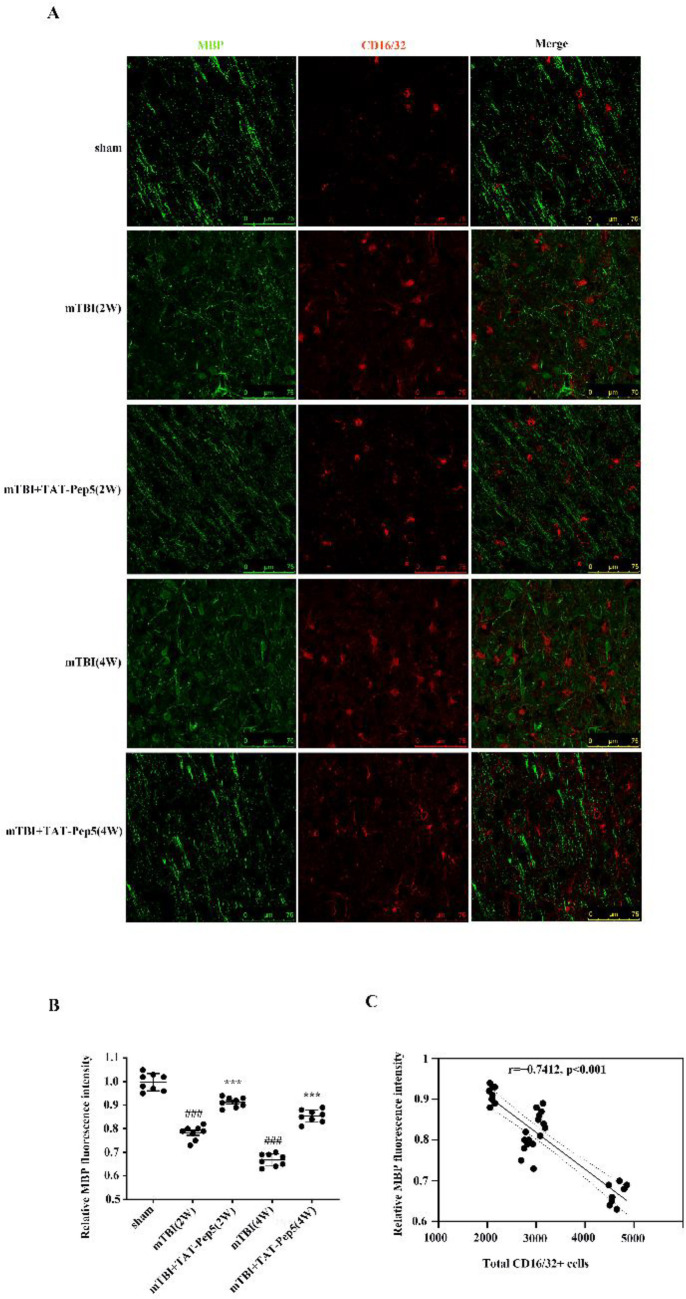
Inhibition of p75NTR alleviated myelin damage after mTBI. **(A)** Representative images of MBP (green) and CD16/32 (red) staining cells in injured brains treated with vehicle or TAT-Pep5 at 2- and 4-weeks post mTBI. Scale bars: 75 μM. **(B)** MBP fluorescence intensity decreased in white matter surrounding the injured cortex from 2 to 4 weeks after mTBI and was reduced by TAT-Pep5 treatment. **(C)** Pearson correlation between MBP staining intensity and number of CD16/32^+^ cells. Data were expressed as mean ± SEM (*n* = 8/group, ^***^p < 0.001 versus vehicle, ^###^*p* < 0.001 versus sham).

In this study, we employed immunofluorescence staining of myelin basic protein (MBP) to observe the myelin damage after mTBI. Our findings demonstrated that mTBI resulted in an apparent reduction in MBP fluorescence intensity at 2 weeks, which was further aggravated at 4 weeks post mTBI (approximately 21.6 and 33.1% reduction compared to sham at 2 and 4 weeks respectively), indicating the persistent myelin damage condition (Figures 5A,B). p75NTR-specific antagonist TAT-Pep5 treatment notably increased MBP fluorescence intensity at 2 and 4 weeks post mTBI (Figures 5A,B). These data suggested that p75NTR blockade alleviated myelin damage after mTBI. The intensity of MBP staining exhibited an inverse correlation with the number of CD16/32 staining cells (Figure 5C, *r* = −0.7412, *p* < 0.001), suggesting a close relationship between demyelination and M1 type microglia activation.

### Inhibition of p75NTR improved neuronal outcomes after mTBI

**Figure 6:**
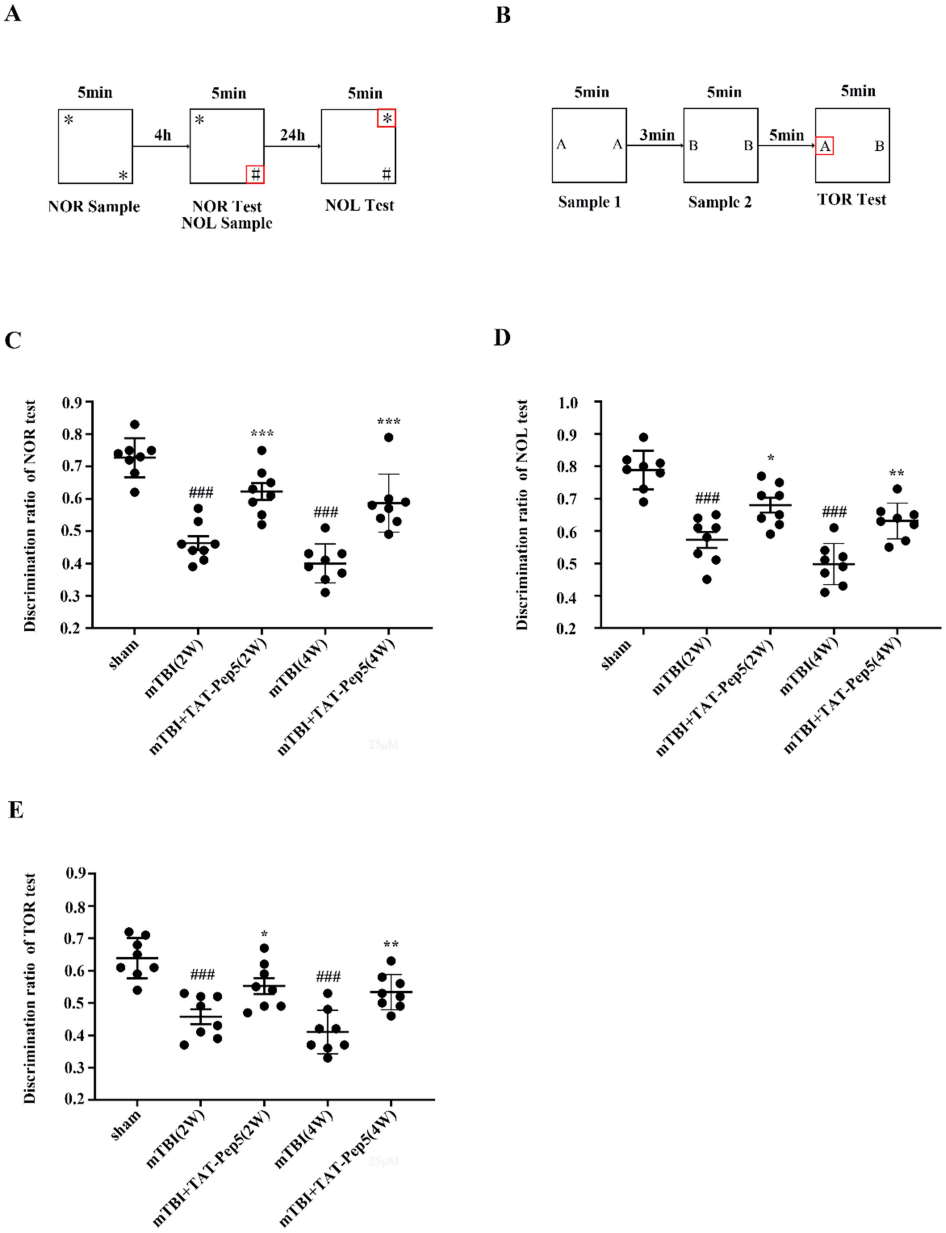
Inhibition of p75NTR improved neuronal outcomes after mTBI. **(A)** Schematic of object recognition tasks. NOR tested short-term memory by replacing an object (*) with (#) after a 4-h delay. NOL tested long-term memory by shifting the position of the familiar object (*) after a 24-h delay. **(B)** TOR tested working memory by presenting pairs of objects. **(C–E)** Discrimination ratio of NOR, NOL, and TOR tests decreased at 2 and 4 weeks after mTBI and was improved by TAT-Pep5 treatment. Data were expressed as mean ± SEM (*n* = 8/group, ^***^p < 0.001 versus vehicle, ^###^*p* < 0.001 versus sham).

The NOR paradigm is consistently reported to be impaired by TBI (Baratz et al., 2015). As expected, NOR test showed a significant difference by treatment group factor (mTBI vs. sham) in discrimination ratio (*p* < 0.001) but not by time factor (2 vs. 4 weeks; *p* > 0.05) (Figure 6C). The NOR results indicated persistent impairment in cognitive function relating to short-term recognition memory following mTBI. A similar trend by treatment group factor was seen in NOL and TOR tests (*p* < 0.001) (Figures 6D,E), suggesting mTBI induced long-term spatial and temporal working memory damage. P75NTR-specific antagonist TAT-Pep5 significantly ameliorated cognitive deficits, as demonstrated by the elevated discrimination ratio in these tests at each timepoint (NOR: *p* < 0.001 at 2 weeks, *p* < 0.001 at 4 weeks; NOL: *p* < 0.05 at 2 weeks, *p* < 0.01 at 4 weeks; TOR: *p* < 0.05 at 2 weeks, *p* < 0.01 at 4 weeks) (Figures 6C–E).

## Discussion

This study investigated the expression of p75NTR in resident microglia following mTBI and its regulatory effects on M1-type microglial activation as well as neuroinflammation-related myelin damage, revealing its pivotal role in mTBI-related pathological processes.

We first quantified the infiltrated peripheral leukocyte and residential microglia in the brain after mTBI by using a CX3C-chemokine receptor 1 (Cx3cr1) and chemokine receptor 2 (Ccr2) double transgenic reporter mouse model. As expected, peripheral leukocytes did not enter the CNS due to the lower severity of BBB damage caused by primary or secondary injury following mTBI, suggesting the residential microglia was the main immune cell associated with mTBI-induced neuroinflammation.

However, considering the systemic inflammatory response following mTBI may last up to 12 months in clinical studies (Visser et al., 2022), we could not exclude the possibility of peripheral leukocyte infiltration over a longer period of time because of the further damage caused by the long-lasting neuroinflammation to the BBB.

Our findings demonstrated p75NTR expression was notably increased in M1-type microglia following mTBI. Blockading of p75NTR tipped the suppression of mTBI-induced microglial activation towards the pro-inflammatory M1-like phenotype, which was accompanied by a reduction in pro-inflammatory cytokines (TNF-α, IL-1*β*, and iNOS), less myelin damage, and a significant improvement in trauma-induced cognitive deficits. These findings suggested that p75NTR might serve as a promising therapeutic target for secondary myelin damage and associated cognitive impairment in the pathophysiological progression of mTBI. This was also the first report about resident microglial expression of p75NTR after mTBI. The expression of p75NTR on resident microglia after TBI might vary depending on the severity of the brain trauma. Some researchers have reported that p75NTR was significantly expressed on peripheral blood-derived pro-inflammatory monocytes/macrophages but not on the activated resident microglia after TBI (Lee et al., 2016; Delbary-Gossart et al., 2016), which was inconsistent with our findings. The inconsistency in findings may stem from the fact that mTBI usually causes minor localized brain tissue damage accompanied by a relatively mild inflammatory response, during which p75NTR may function as an inflammatory regulatory molecule involved in microglial activation and response. In contrast, moderate to severe injuries, characterized by extensive tissue damage, trigger a robust immune response, potentially leading microglial cells to enter an “overactive” state and exhibit “immune suppression,” which results in less pronounced upregulation of p75NTR expression to avoid microglial overstimulation. In our study, by using immunofluorescence to co-label CD206, CD16/32, and p75NTR, we noted p75NTR was upregulated only in M1 cells, while no upregulation was observed in M2 cells post-mTBI, indicating that p75NTR was mainly involved in pro-inflammatory responses rather than repair processes. The expression of p75NTR restricted to M1-type microglia suggested that p75NTR might mediate the polarization of microglia through cell subtype-specific signaling regulatory mechanisms incited by distinct phases or microenvironments of neuropathological process associated with mTBI. Although the underlying molecular mechanisms are not accurately defined, a few studies have hinted at the possible roles of trans-acting transcription factor 1 (Sp1), which binds to the multiple Sp1 binding sites assembled in p75NTR proximal promoter to induce p75NTR expression (Sehgal et al., 1988; Patil et al., 1990). Sp1 expression is increased in M1-type microglia spinal cord cells after mechanical injury, where it promotes M1 polarization of microglia and inhibits microglia polarization from M1 to M2 phenotype (Guo et al., 2024; Xu et al., 2024).

Although neuroinflammation is believed to be an important secondary physiological response to TBI, most of the current knowledge on this response is derived from research into moderate and severe TBI. Similarly, demyelination resulting from oligodendrocyte loss is mostly studied in moderate and severe TBI and has rarely been explored in the mTBI context. Here we offered data showing an inverse correlation between demyelination and M1 type microglia activation, which could help us to alleviate the disturbance caused by chronic cognitive impairment following mTBI.

One interesting phenomenon found in our study was that the most increased CD16/32 and CD206-positive cells were also Trem2 positive after mTBI. Trem2 is a key molecule regulating the transition of microglia to the DAM state, playing a vital role in various neurodegenerative diseases (Rangaraju et al., 2018). In AD pathology, TREM2 is found to be involved in microglial survival, proliferation, clustering around amyloid-beta (Ab) plaques, phagocytosis, and metabolism (Yeh et al., 2017). Indeed, recent epidemiology studies have indicated that TBI can increase the risk of developing neurodegenerative diseases such as AD (Washington et al., 2016). Compelling studies have supported the hypothesis that mTBI accelerates the formation and accumulation of Amyloid-β (Yang et al., 2015; Grant et al., 2018). Although the research that reported these findings had limitations, particularly regarding mTBI patients, these findings could link TBI with AD, suggesting that mTBI might lead to long-term cognitive impairment through the pathophysiological procedures with AD features and reinforce the idea that mTBI might activate Trem2+ microglia with DAM characteristics. Whether Trem2+ microglia in mTBI responds to Amyloid-β as it does to AD is not clear, but the p75NTR inhibition notably reduced the numbers of Trem2-positive M1 type microglia, suggesting a potential interaction between p75NTR and Trem2 in regulating microglial activation. While the precise molecular interaction mechanisms between these two receptors remain to be fully elucidated, these findings provide novel insights into the pathogenesis of post-mTBI cognitive impairment and highlight the therapeutic potential of dual-targeting p75NTR and Trem2 signaling pathways.

This study focused on assessing microglia activation and cognitive function 2 to 4 weeks after mTBI. While these time points provided insights into the acute and subacute phases of recovery, with the advantage of excluding possible interference caused by mTBI-induced neurodegeneration, the study lacks long-term observations. Cognitive impairment from mTBI may continue to progress over a longer period. Therefore, future research should prioritize longer observation periods (e.g., 3 months or more) to evaluate the persistence of therapeutic effects and the recovery process.

In conclusion, this study elucidated the critical role and molecular mechanisms of p75NTR in mTBI-induced neuroinflammation and cognitive impairment. By establishing an mTBI mouse model, we demonstrated that upregulated p75NTR expression triggered neuroinflammatory responses through the activation of DAM-like (Trem2-positive) M1 microglia. Importantly, targeted inhibition of p75NTR using the antagonist TATpep5 effectively enhanced CNS repair and restored learning-memory functions.

## Data Availability

The original contributions presented in the study are included in the article/Supplementary material, further inquiries can be directed to the corresponding author.

## Supplementary material

SUPPLEMENTARY FIGURE 1 CD206+ M2 phenotype microglia did not express P75NTR and Trem2 after mTBI. **(A)** Representative images of p75NTR (green) and CD206(red) staining cells in injured brains at 2 weeks post mTBI. Scale bars: 8 μM. **(B)** Representative images of CD206(green) and Trem2(red) staining cells in injured brains treated with vehicle or TAT-Pep5 at 2- and 4-weeks post mTBI. Scale bars: 25 μM. **(C)** The percentage of Trem2+ cells in CD206+ microglia with or without TAT-Pep5 treatment. Data were expressed as mean ± SEM (*n* = 8/Group). **(D)** CD206+/Trem2+ cells increased in white matter surrounding the injured cortex from 2 to 4 weeks after mTBI. TAT-Pep5 treatment did not significantly change the number of CD206+/Trem2+ microglial cells. Data were expressed as mean±SEM (*n* = 8/Group, $ *p* > 0.05 versus vehicle, ###*p* < 0.001 versus sham).

SUPPLEMENTARY FIGURE 2 Full-length Western blot and gels of Figure 4E.
