# Supplementary figures and images for: P75NTR blockading inhibits Trem2+ M1 phenotype microglia activation and myelin damage following mild traumatic brain injury
Source: Front Neurosci. 2026 Jan 7;19:1641112. doi: 10.3389/fnins.2025.1641112 (PMC12819785; doi:10.3389/fnins.2025.1641112)

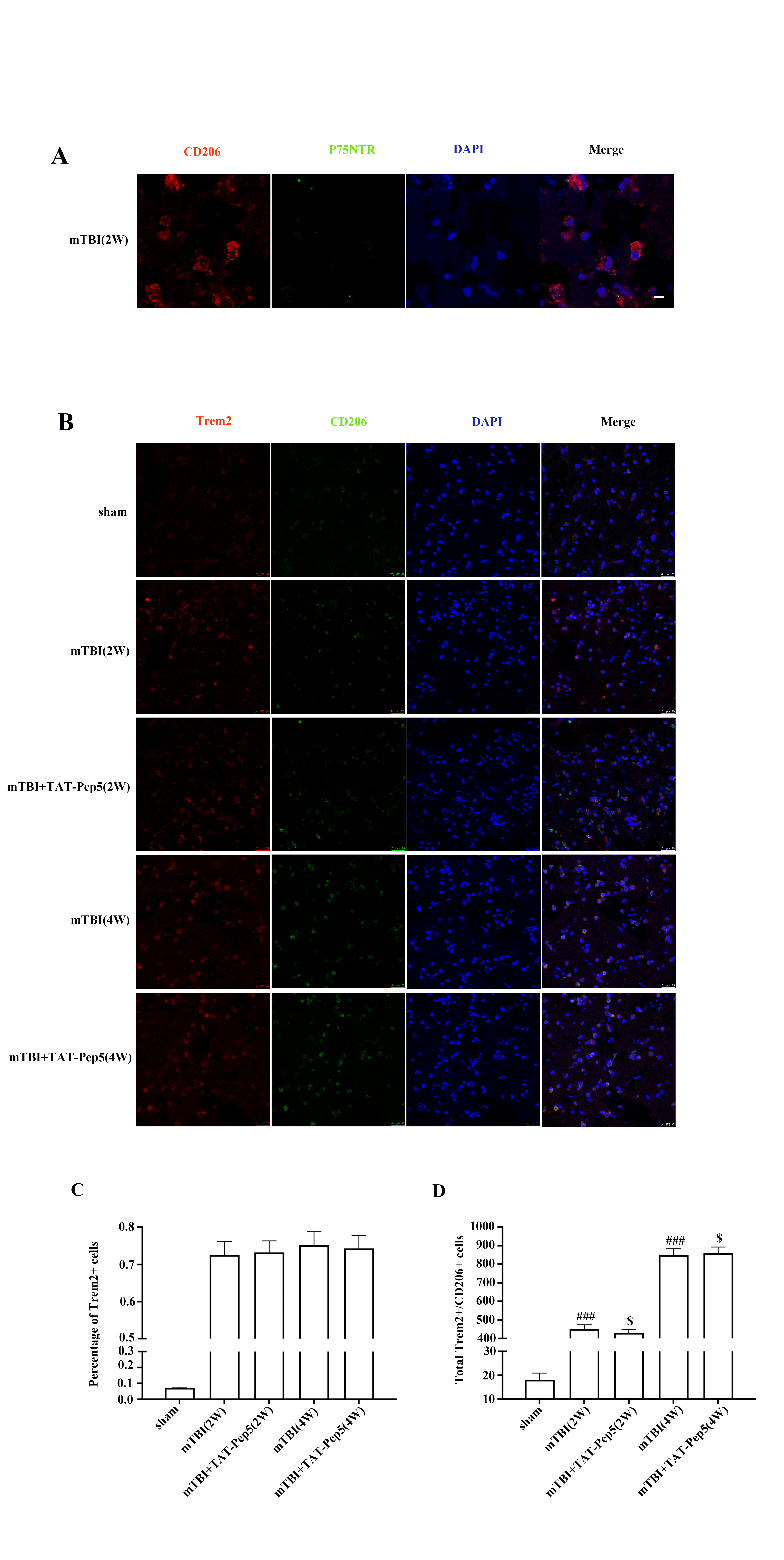

Supplement: Supplementary file 1 [file Image_1.tif]

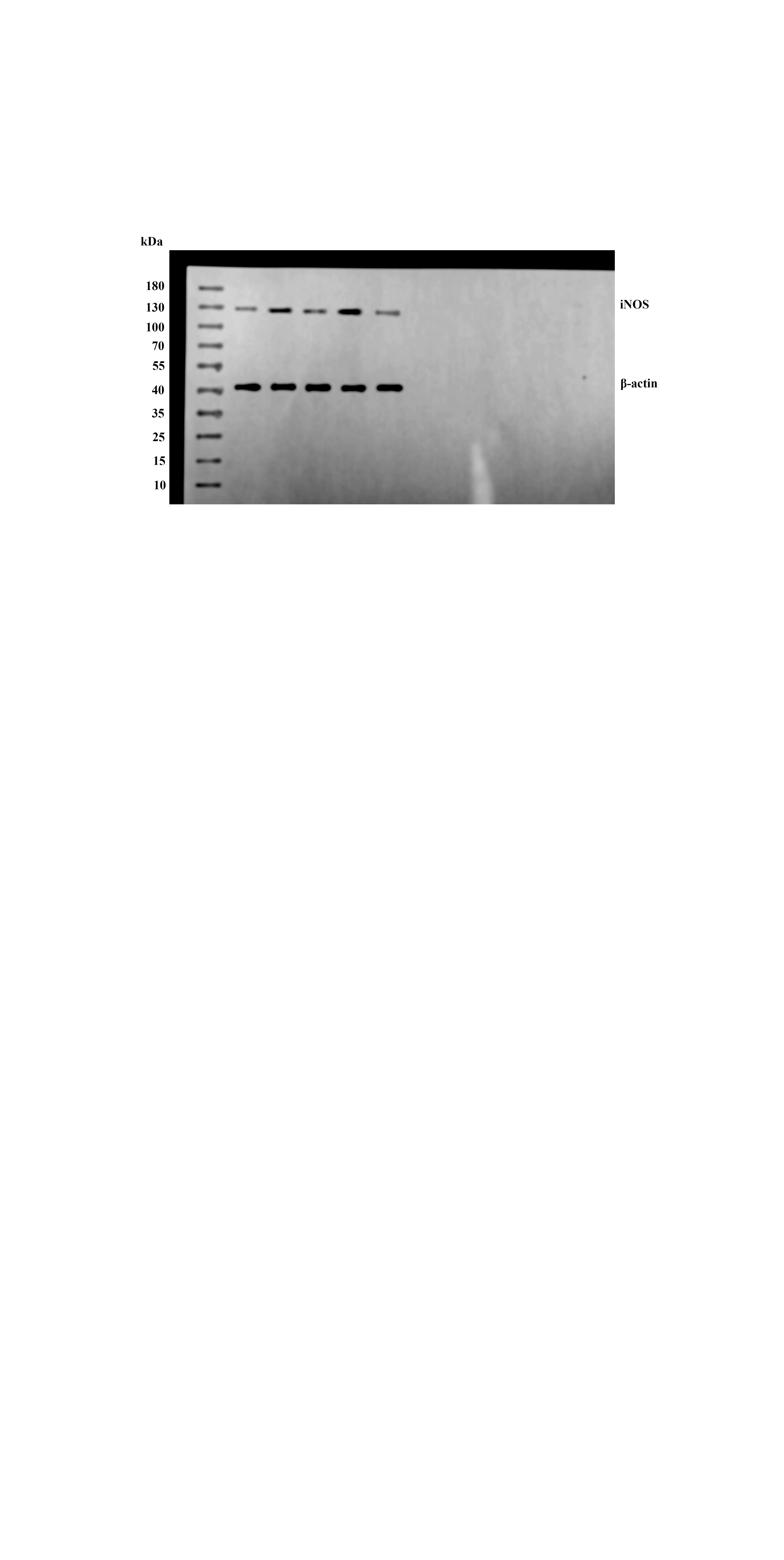

Supplement: Supplementary file 2 [file Image_2.tif]
